# Online Interventions to Improve Mental Health of Pediatric, Adolescent, and Young Adult Cancer Survivors: A Systematic Review and Meta-Analysis

**DOI:** 10.3389/fpsyt.2021.784615

**Published:** 2021-12-23

**Authors:** Nutthaporn Chandeying, Therdpong Thongseiratch

**Affiliations:** ^1^Department of Obstetrics and Gynecology, Faculty of Medicine Vajira Hospital, Navamindradhiraj University, Bangkok, Thailand; ^2^Department of Pediatrics, Faculty of Medicine, Prince of Songkla University, Hat Yai, Thailand

**Keywords:** cancer survivors, online, internet, AYA, pediatric, children, mental health

## Abstract

**Objective:** Over the last 10 years, online interventions to improve mental health have increased significantly. This study's primary objective was to determine the effectiveness of online interventions in improving the mental health of pediatric, adolescent, and young adult (PAYA) cancer survivors. The secondary objective was to identify the independent variables associated with online intervention efficacy for mental health improvement.

**Methods:** On June 25–30, 2021, we searched the Medline, PsycINFO, EMBASE, and Cochrane databases for eligible English language publications that reported randomized controlled trials of online interventions aimed at improving mental health among PAYA cancer survivors. The results were analyzed using a systematic review and a three-level meta-analysis.

**Results:** Thirteen studies met the inclusion criteria. In six (42%) studies, the intervention focused on physical activity enhancement, while ten (77%) studies used self-directed interventions. Online interventions were more efficacious, compared to control conditions, in improving sleep *g* = 0.35 (95% CI 0.04–0.66) and psychological well-being *g* = 0.32 (95% CI 0.09–0.56), but not for reducing the symptoms of depression *g* = 0.17 (95% CI −0.13 to 0.47), anxiety *g* = 0.05 (95% CI −0.15 to 0.25), and pain *g* = 0.13 (95% CI −0.13 to 0.39).

**Conclusion:** Online interventions were generally effective in improving mental health in PAYA cancer survivors, although negative results were found in some critical outcomes. More high-quality evidence is needed for definite conclusions to be drawn. The study protocol was registered in PROSPERO (CRD42021266276).

## Background

Advanced treatment modalities have increased the survival rate of children, adolescents, and young adults with cancer to above 80% (1, 2). The United States has over 400,000 cases, and this population is growing, with 10,000 new cases diagnosed each year (3). Centers for Disease Control and Prevention have defined that cancer survivor is anyone who has been diagnosed with cancer, from the time of diagnosis through the balance of his or her life (4). A cancer diagnosis during children (defined as ages 0–15) and adolescence or young adulthood (AYA; defined as ages 15–39) generates unique medical and psychological needs as developmental milestones are simultaneously impacted (5). For instance, a critical element of the adolescent population (i.e., those aged 15–20) is the confluence of the cancer experience with a period of rapid biological and psychological changes. Specifically, this group may face difficulties with autonomy and independence, sexual and romantic maturity, reproduction, and economics (6). Additionally, adverse effect of cancer therapy can have a physical impact on survivors, as well as affect their self-image and well-being in the short and long term (5, 6). Consequently, pediatric, adolescent, and young adult (PAYA) cancer survivors face a variety of problems, including disruptions in education, careers, and social milestones, as well as the long-term side effects of their treatments (7–9).

Cancer treatment complications and unique psychological development combine to associated with mental health problems (10), including post-traumatic stress disorder (11), anxiety (12), and depression (13), which are frequently observed in PAYA cancer survivors. More than half of PAYA cancer survivors reported at least one significant chronic mental health problem that requires ongoing care (14). However, they are often lost to follow-up (15, 16) and have an inadequate understanding of their cancer care (17). Therefore, more research on PAYA cancer survivors' mental health interventions to solve unmet psychological needs is critically needed.

Cancer knowledge, self-efficacy, coping style, and physical activity play a significant role in the onset and persistence of mental health problems in PAYA cancer survivors (18).

Having a limited or inaccurate cancer knowledge may impair survivors' ability to communicate effectively with healthcare providers, which may have an effect on the quality of care they receive. Inadequate cancer knowledge also prevents survivors from taking critical efforts to avoid health risks in their daily lives (e.g., smoking cessation, physical activity, diet) (19, 20). Cancer-related self-efficacy refers to the belief that a survivor can successfully carry out the behaviors necessary to achieve the desired outcome in connection to the effects of cancer and its treatment (21). Self-efficacy is connected with greater self-care behaviors and lower physical and psychological symptoms in cancer patients (22). For the coping style, while some cancer survivors employ adaptive coping methods to alleviate suffering, others rely on less adaptive coping mechanisms (23). Poor adaptive coping may provide a momentary reprieve from anxiety-provoking thoughts, these ideas may grow more obsessive and intrusive with time (24). Physical activity has been shown to be beneficial in reducing the cluster of symptoms associated with cancer treatment (25). Regular physical activity or exercise has been shown to promote positive psychological functioning (26) and can be used to cope with the side effects of cancer and its treatment, including increased feelings of depression, anxiety, sleep difficulties, and cancer-related fatigue (27), as well as the cognitive confusion or impairment that frequently persists following cancer treatment (28, 29). A bidirectional relationship between these factors and mental health outcomes has been proven (30). From this perspective, intervention programs seek to improve PAYAs' mental health by increasing their cancer knowledge, self-efficacy, coping style, and physical activity (30).

Despite the well-established interventions for PAYAs' mental health, few PAYA cancer survivors receive such interventions (31). Several challenges faced by PAYA cancer survivors when using mental health services include the cost of services, inconvenient timing or location, and a shortage of competent staff (32–34). Technology based interventions, using the webpages, email, mobile applications, and social media can help them to overcome these constraints (35). Technology-based interventions are those that use a technological platform to give information, support, and therapy for physical or mental health problems (36). Early technology-based interventions in the field of mental health were typically static with minimal interactivity and were delivered offline (e.g., via a PC or laptop equipped with a CD-ROM or installed software), requiring patients to be in a certain location to receive the intervention. Recent advancements in digital technology have enabled the potential of online intervention, which is typically defined as the delivery of a computerized program via the Internet (37). Online interventions utilize telecommunications systems (e.g., text messaging, emailing, and videoconferencing) to provide the distant delivery of synchronous and asynchronous interventions (36–38). PAYA cancer survivors may participate in interventions from the convenience of their own residents and at a lower cost than that for face-to-face interventions, depending on the quality of technology-based interventions (39, 40). The most common manner to offer technology-based interventions is through online platforms (38, 41). The benefits of these interventions include immediate access, the ability to update content easily, the patients' ability to ask questions and receive help, and the ability to track their progress (42, 43). Because internet penetration is constantly expanding, online interventions for improving mental health of PAYA cancer survivors are increasingly promising (44).

Both single studies (45, 46)and two meta-analyses studying on the effect of distance-delivered physical activity interventions and technology-assisted psychosocial interventions support the efficacy of online interventions on improving PAYA's mental health (47, 48). However, a recent meta-analysis studying on the effect of digital self-management interventions revealed an inconsistent finding (49). Mizrahi et al. (47) demonstrated the moderate effectiveness of distance-delivered physical activity interventions in improving psychosocial outcomes in childhood cancer survivors. Zhang et al. (48) discovered that technology-assisted interventions are effective for a variety of children's outcomes (distraction from intrusive treatment—medium effect size, mental health—small effect size, physical health—small effect size, and cancer knowledge—small effect size). However, Hong et al. (49) found that digital self-management interventions do not influence the quality of life and physical activity.

Apart from drawing inconclusive findings, these meta-analyses have several noteworthy limitations. Overall, previous meta-analyses examined the effect of technology-based interventions without examining the effectiveness of online interventions separately. For example, Zhang et al. (48) conducted a meta-analysis on the effectiveness of technology-assisted psychosocial interventions for childhood, adolescent, and young adult cancer survivors and found that eight out of twenty-eight randomized controlled trials (RCTs) examined online interventions; however, their independent effectiveness was not examined. The effect sizes for mental and physical health outcomes were pooled by merging research with a variety of objectives. Mizrahi et al. (47) conducted a meta-analysis, which included four trials that focused exclusively on distance-delivered physical activity interventions for childhood cancer survivors. Hong et al. (49) focused exclusively on physical health outcomes and quality of life. No study analyzed mental and physical health outcomes separately.

The primary objective of this study was to determine the effectiveness of online interventions in improving the mental health of PAYA cancer survivors. The secondary objective was to identify the independent variables associated with online intervention efficacy for mental health improvement. This study included difference instruments and outcomes. Furthermore, several groups explored the use and effects of online intervention and reported meaningful results. To date, evidence regarding the effects of online intervention on mental health of PAYA cancer survivors has not been synthesized for clinical practice.

## Methods

### Protocol

This systematic review and meta-analysis followed the Preferred Reporting Items for Systematic Reviews and Meta-Analyses (PRISMA) guidelines (50). The study protocol was registered in PROSPERO (CRD42021266276).

### Selection Procedure

English language reports published in peer-reviewed sources were included. We assessed study eligibility using the PICO approach (population, intervention, comparison, and outcome) (51).

#### Population

Children, adolescents, or young adult patients with or survivors of cancer (0–39 years of age with cancer diagnoses) (48). Studies of adult patients with cancer (>40 years of age with cancer diagnoses), patients without current cancer or a cancer history, or caregivers of patients with cancer were excluded.

#### Intervention

Any psychological intervention that were delivered online, using a computer or a mobile application (both therapists delivered and self-directed). Interventions that involved physical approaches—for example, physical activity intervention—could be included in the intervention but only if they were delivered online. Interventions were not required to directly target mental health.

#### Comparison

Eligible studies were required to use a control group—for example, waitlist, treatment as usual, or alternative control. Case studies, studies that included only two active psychological interventions and no control group (e.g., non-inferiority trials) were excluded from the meta-analysis.

#### Outcome

Pre- and postintervention data, or pre–post change score data on one or more quantitative mental health outcome. Mental health outcome could be both primary and secondary outcome. Studies that used qualitative assessments, quantitative measures at one-time point only, or only measures of quality of life were excluded. Studies needed to report results as either pre–post means and standard deviation/SE in all groups with sufficient detail to allow the calculation of an effect size, or the data could be requested from the authors. Studies that lacked sufficient data to calculate the effect size or had a sample size of less than *N* = 10 were also omitted, as were pilot studies that did not adequately explore the effectiveness of interventions in improving PAYAs' mental health.

### Searching Strategies

The literature was searched extensively for RCTs examining online interventions that were aimed at improving mental health for PAYA cancer survivors. We searched for studies on June 25–30, 2021, in the Medline, PsycINFO, Web of Science, and Cochrane Library databases. The following keywords were used in the titles and abstracts: participants (e.g., child^*^ OR pediatric OR adolescents^*^ OR young adult OR AYA), cancer (e.g., cancer OR cancer survivor^*^ OR oncology^*^), intervention (e.g., interv^*^ OR program^*^ OR educat^*^ OR Psychosocial^*^), online (e.g., online^*^ OR Internet^*^ OR web^*^), and RCTs (e.g., controlled trial OR trial^*^ OR RCT). We also reviewed the bibliographies of relevant review articles to identify additional publications. Please refer to Supplementary Appendix 1 for all the search strategies and approaches used to locate relevant articles in all the databases.

### Study Selection

RefWorks was used to eliminate duplicate data. Both authors independently reviewed the studies' titles and abstracts for compliance with the inclusion criteria. Thereafter, the same authors reviewed the papers that were considered for full-text screening individually, and any disagreements were handled through a discussion. The inter-reviewer reliability (Kappa) was 0.93 (*p* < 0.001), indicating good inter-reviewer agreement.

### Data Extraction

We extracted the studies' identifying data (i.e., authors, publication year, and country) and the data necessary for the effect size calculation (i.e., sample sizes, means, and standard deviations). Similarly, data on study procedures, interventions, and sample characteristics were retrieved. All extracted data were imported into R version 4.1.0, which was used to conduct the analyses.

### Analyses and Coding of Independent Variables Associated With Intervention Efficacy

Consistent with our research question, the primary outcome was the mental health of PAYA cancer survivors, which was measured using tools with established psychometric properties. The secondary outcomes were independent variables that were associated with online intervention efficacy for mental health improvement. In the sensitivity analyses, the participants' ages were classified as follows: children and adolescents (<20 years) and adults (≥20 years) (30). Mental health outcomes were classified into five categories: depression, anxiety, pain, sleep, and psychological well-being. Psychological content was classified as follows: cognitive behavioral therapy (CBT), psychoeducation, physical activity, and legacy intervention (30, 47, 48). We also coded online interventions as individual and group deliveries. The comparison group comprised two conditions: a waiting list and an alternative treatment (face-to-face counseling or offline self-guided interventions). If patients underwent the intervention independently, they were classified as self-directed; they were classified as therapist-involved if the intervention program was administered directly by a therapist (e.g., videoconference) (30, 47, 48). Additionally, the online platforms for each intervention module were coded using the data contained in the articles. We coded platforms as websites alone, websites with text messages, wearable respiratory monitoring and applications, wearable physical activity monitoring and social media applications, chatbots, and VDO conferences (47–49). The continuous factors considered were the participants' mean age, length of the intervention, dropout rate, and bias risk.

### Meta-Analytical Procedure

Because the studies were conducted in different countries and with participants from diverse socioeconomic backgrounds, the effect size was determined using a random-effects model (52). At each time point, the effect size on PAYAs' mental health was calculated independently (posttest and follow-up). The effect sizes of the online interventions were assessed in comparison to the control condition. Sensitivity analyses were employed to account for clinical and methodological heterogeneity in the secondary objective (53). The continuous variables were analyzed using meta-regression (54).

### Effect Size Calculation

Compared to the control condition, the standard mean difference (SMD) as well as 95% confidence interval (CI), as the measure of online intervention effect, were calculated and then converted into the adjusted Hedges' *g* (55). The effect size is small between 0.20 and 0.50; medium between 0.50 and 0.80; and large >0.80 (56). SMD is the difference in means between the online intervention and comparison groups divided by the pooled SD of both groups. A positive effect size means that the intervention group outperformed the control group in improving mental health.

A three-level meta-analysis was used since the mental health outcomes were measured using several instruments (in the same study) (57). For each effect size, Level 1 reflects the sampling variance. The variance between the effect sizes within a study was assumed to be at Level 2. Level 3 illustrates the variation in effect sizes among the studies. By fitting meta-analysis models without an intercept, we quantified the extent of the intervention effect. Statistical analysis was performed to examine the variables, both within and between studies (58).

The *I*^2^ statistic was used to quantify heterogeneity. This statistic indicates the percentage of observed variance (*I*^2^ = 0 indicates no heterogeneity; *I*^2^ = 25 indicates low heterogeneity; *I*^2^ = 50 indicates a medium heterogeneity; *I*^2^ = 75 indicates a high heterogeneity) (59). Because a basic assumption of publication bias test (e.g., funnel plots, Egger's test) is the independence of effect sizes, we were unable to assess publication bias for this meta-analysis. The conventional test of publication bias was not applicable since we included all relevant effect estimates from each study (60).

### Quality Assessment

The quality of the studies was determined by using the Cochrane Collaboration's risk of bias RoB 2.0 tool to calculate the risk of bias (ROB) in randomized trials. ROB was assessed across five domains (52). Each of the five domains was assigned a low or high risk of bias as well as some concern risks. Both authors individually examined the probability of ROB, with any disagreements being discussed. The kappa coefficient was used to determine the inter-rater agreement between the authors (50).

## Results

### Included Studies

**Figure 1:**
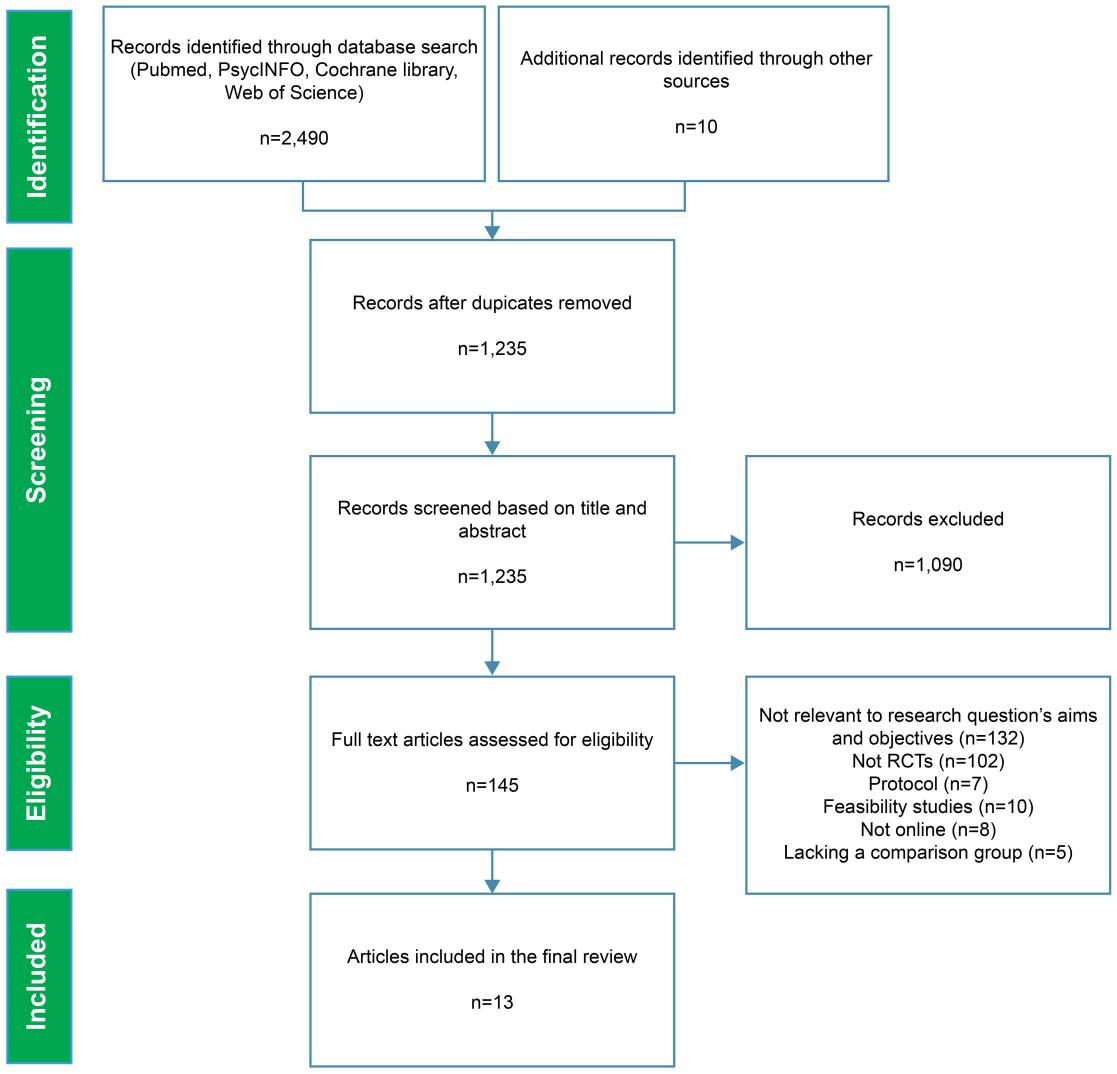
PRISMA flow diagram.

The PRISMA flow diagram is shown in Figure 1. The search yielded 2,490 papers, with ten additional records discovered by reviewing the references of the systematic reviews that were included, and 1,265 duplicates were excluded. The remaining studies (*n* = 1,235) were screened using the keywords in titles and abstracts, which eliminated 1,090 more studies. The full text of the remaining 145 studies was then evaluated, with 132 studies being eliminated for the following reasons: 102 studies were not RCTs; 17 studies dealt with protocols, usability, or feasibility; eight studies did not deliver online; five studies did not have a control group or a comparison intervention. Finally, 13 studies were included in this systematic review.

### Systematic Review

#### Sample Description

All online interventions for PAYA cancer survivors that were included focused on multiple cancer types. The sample sizes ranged from 38 to 150 subjects (*M* = 71.85, SD = 37.73). Participants' mean age ranged from 10.6 to 44.1 (*M* = 23.73, SD = 9.30). Most studies (*n* = 11) were developed in the US, and the rest in China and Australia. We found that 46.2% of the studies aimed at PAYA cancer survivors' general mental health prevention (*n* = 6) (61–66). In contrast, 53.8% of the studies focused on the participants' physical health (*n* = 7) (67–73), including physical activity enhancement (*n* = 5; 38.5%) (68, 69, 71–73), sleep (*n* = 1; 7.7%) (70), and chronic pain (*n* = 1; 7.7%) (67). Finally, we found a dropout rate of 1.5–40%, with a mean of 15.65% (SD = 13.21).

#### Study Characteristics

Table 1 lists the characteristics (e.g., age, format, outcomes) of the included studies. Online intervention for childhood, adolescent, and young adult cancer survivors was compared to an alternative treatment (*n* = 7; 53.8%), a treatment as usual (*n* = 3; 23.1%), and a waiting list (*n* = 3; 23.1%). Almost all interventions were primarily self-directed, with or without minimal therapist support. Therapist-led interventions (*n* = 1) were compared to an alternative treatment in one study (66). The majority of the studies (*n* = 10) were two-arm randomized trials, with three being three-arm randomized trials. The first three-arm randomized trial compares the two online interventions to an alternative treatment. The second compares online intervention to a waiting list as well as an alternative treatment. The final one compares online intervention with two alternative treatments. Among the included studies, 76.9% (*n* = 10) show two measurement time points of the outcomes (baseline and post-test), whereas 23.1% (*n* = 3) have at least one additional follow-up assessment.

**Table 1:** Characteristics of online interventions for pediatric, adolescent, and young adult cancer survivors.

**References**	**Country**	**N**	**Mean age (range)**	**Intervention**	**Format**	**Comp**	**Length**	**Follow-up**	**Outcomes**	**Risk of bias**
Akard et al. (61)	US	150	10.6 (7–17)	Legacy intervention	I/W	WL	2 w.	–	PedQL	High
Albert et al. (67)	US	65	44.1 (18–50)	Respiratory monitoring and feedback	I/WD	WL	30 d.	–	BPI GAD-7 PHQ-8 PROMIS SF-12	High
Berg et al. (62)	US	63	32.5 (18–50)	Hope-based intervention (CBT)	I/A	AT	8 w.	6 m.	SF-36 FACT-G PHQ-9	High
Casillas et al. (63)	US	78	21.0 (11–39)	Psychoeducation	I/W/T	AT	8 w.	–	SCAM	High
Greer et al. (64)	US	45	25.0 (18–29)	Vivibot chatbot (Psychoeducation)	I/S	WL	4 w.	–	PROMIS DES	High
Howell et al. (68)	US	97	12.7 (11–15)	PAE	I/W	AT	24 w.	–	PedQL	Some concerns
Huang et al. (69)	US	38	13.0 (10–16)	Fit4Life (PAE)	I/W/T	AT	4 m.	–	CDI	Some concerns
Kunin-Batson et al. (65)	US	52	21.0 (15–29)	Psychoeducation	I/W	TAU	12 m.	–	STAI	High
Li et al. (70)	China	143	28.4 (15–39)	PAE	G/WD/S	TAU	8 w.	3 m.	PSQI FACT-G	High
Mendoza et al. (71)	US	59	16.6 (14–18)	PAE	G/WD/S	TAU	10 w.	–	PedQL PedQL-C	Low
Rabin et al. (72)	US	18	32.2 (18–39)	PAE	I/W	AT	12 w.	–	POMS	High
Sansom-Daly et al. (66)	Australia	40	20.6 (15–25)	VDO Conference CBT	G/V	AT	6 w.	12 w. 12 m.	DASS-21	High
Valle et al. (73)	US	86	30.8 (21–39)	FITNET (PAE)	G/WD/S	AT	12 w.	–	FACT-G	Some concerns

*Comp, comparison condition; AT, alternative treatment; TAU, treatment-as-usual; WL, waiting list; Intervention: CBT, cognitive-behavior therapy; PAE, physical activity enhancement; Format: I, individual; G, group; W, website; A, Application; T, text massage; WD, wearable device; S, social media; V, VDO conference; Length: d, days; w, weeks; follow-up: m, months*.

*See online Supplementary Appendix 2 for references*.

#### Intervention Characteristics

In total, 46.2% (*n* = 6) of the studies used psychological interventions (e.g., psycho-education, CBT, and legacy intervention) to improve mental health. Another six studies (*n* = 6) provided a physical activity enhancement program, while another study provided respiratory monitoring and feedback (*n* = 1). The median treatment duration was 8 weeks. Psychoeducation took an average of 8 weeks (SD = 3.3), CBT took 7 weeks (SD = 1), and physical activity enhancement programs took 13 weeks (SD = 5.2).

In total, 69.2% (*n* = 9) of the included studies used an individual online delivery format. Most of the interventions (*n* = 6) used a web-based intervention, two studies (*n* = 2) used web-based intervention with additional text messages, and four studies (*n* = 4) used web-based intervention without interactive contact. One study (*n* = 1) used a mobile application, one (*n* = 1) used a wearable respiratory monitoring device, and one (*n* = 1) used a social media chatbot. The other studies (*n* = 4) had a group online format that used wearable devices with social media (*n* = 3) and videoconferences (*n* = 1). In total, 76.9% (*n* = 10) of the trials were self-administered, whereas 23.1% (*n* = 3) included therapist support via videoconference (*n* = 1) and phone calls (*n* = 2).

#### Outcome Measured

We found some diversity in the measures used to assess online interventions for mental health effects. Depression, anxiety, sleep, pain, and psychological well-being were all considered as mental health outcomes. For additional information, see Table 2. All 13 studies used self-reports to assess the effects of the intervention, using a variety of tools. One study considered both self-reported and parent-reported outcomes.

**Table 2:** Instruments for outcomes.

**Outcomes**	**Instruments**	**Subtest**
Depression	Patient Health Questionnaire 8-item (PHQ-8)	
	Patient Health Questionnaire-9 item (PHQ-9)	
	Patient-Reported Outcomes Measurement Information System (PROMIS)	Depression
	Children's Depression Inventory (CDI)	Negative mood, interpersonal problems, negative self-esteem, ineffectiveness, anhedonism
	Depression, Anxiety and Stress Scale 21-item Short Form (DASS-21)	Depression
Anxiety	Pediatric Quality of Life Inventory (PedsQL) Cancer Module	Worry, procedural anxiety, treatment anxiety
	Generalized Anxiety Disorder 7-item (GAD-7)	
	Patient Reported Outcomes Measurement Information System (PROMIS)	Anxiety
	State Trait Anxiety Inventory (STAI)	State, Trait
	Depression, Anxiety and Stress Scale 21-item Short Form (DASS-21)	Anxiety
Sleep	Patient Reported Outcomes Measurement Information Systems (PROMIS)	Sleep disturbance
	Pittsburgh Sleep Quality Index (PSQI)	
Pain	Pediatric Quality of Life Inventory (PedsQL) Cancer Module	Pain
	Medical Outcome Study 36-Item Short Form Health Survey (SF-36)	Pain
	Brief Pain Inventory (BPI)	
Psychological well-being	Medical Outcomes Study Short Form (SF-12)	
	Medical Outcome Study 36-Item Short Form Health Survey (SF-36)	Emotional well-being
	Functional Assessment of Cancer Therapy-General (FACT-G)	Emotional well-being
	Differential Emotions Scale (DES)	Positive emotion, negative emotion
	Pediatric Quality of Life Inventory (PedsQL) version 4.0	Emotional functioning
	Profile of Mood States (POMS)	

#### Risk of Bias

**Figure 2:**
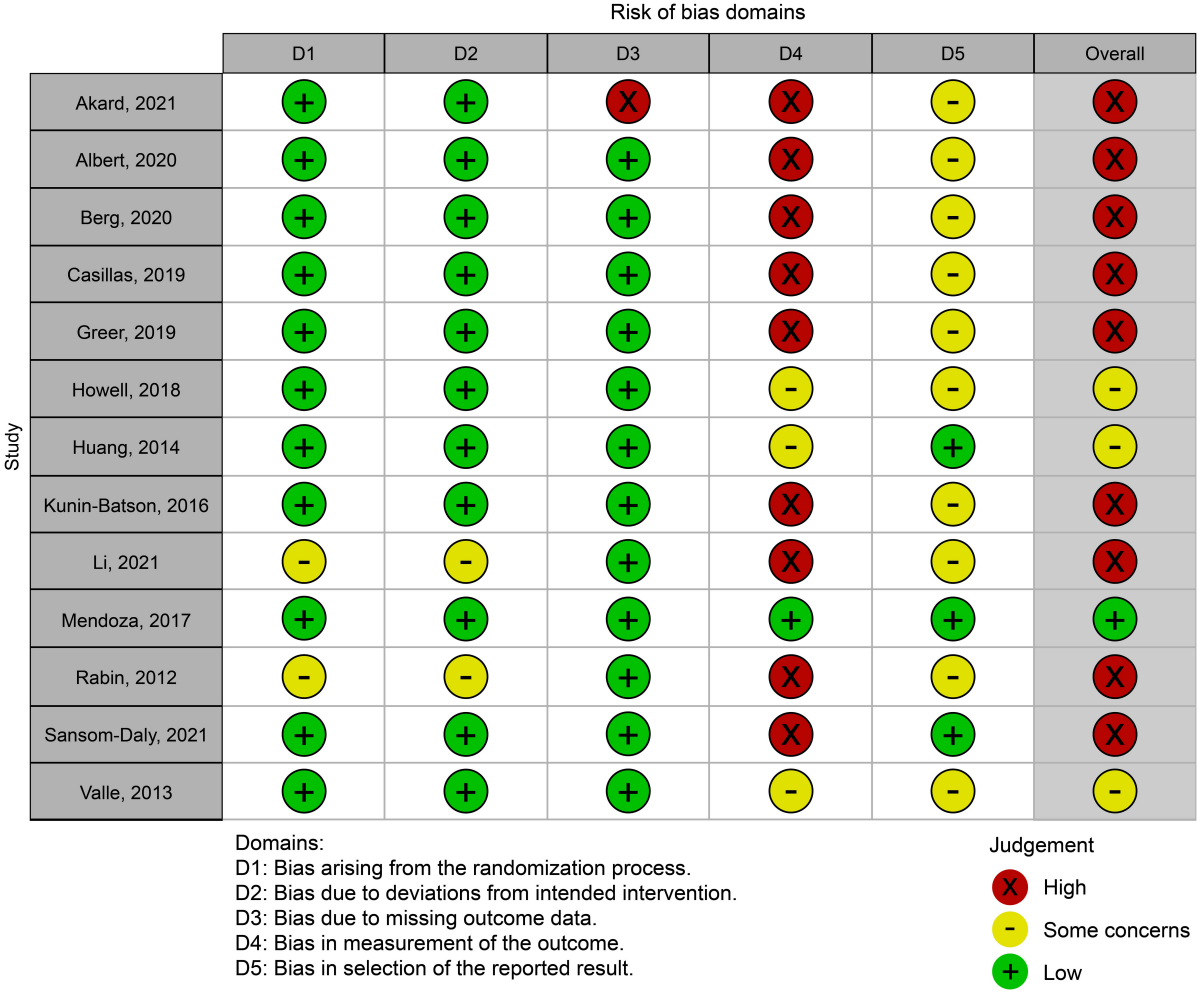
Risk of bias summary.

Figure 2 shows a summary of the authors' ROB judgments for each ROB domain. All included studies were found to have ROB. Kappa was 0.92 (*p* < 0.001), indicating good inter-evaluator agreement. Eleven studies were classified as having low ROB in the randomization procedure domain, while two were rated as having some concern. In the second domain, that is, deviations from intended interventions, 11 studies were rated as having a low ROB rating, while two were rated as having some concern. In the third domain, one study was classified as having a high ROB and missing outcome data, while the others were rated as having a low risk of bias. Only one study was judged as having a low risk of bias in the fourth area—assessment of the outcome—owing to the assessors' lack of blinding. Nine studies were rated as having high ROB, and three were rated as having some concerns. As only three studies had a pre-registered protocol, the fifth domain, selection of the reported results, was graded as having some bias concerns for the other ten studies.

### Meta-Analyses

#### Primary Analysis

**Figure 3:**
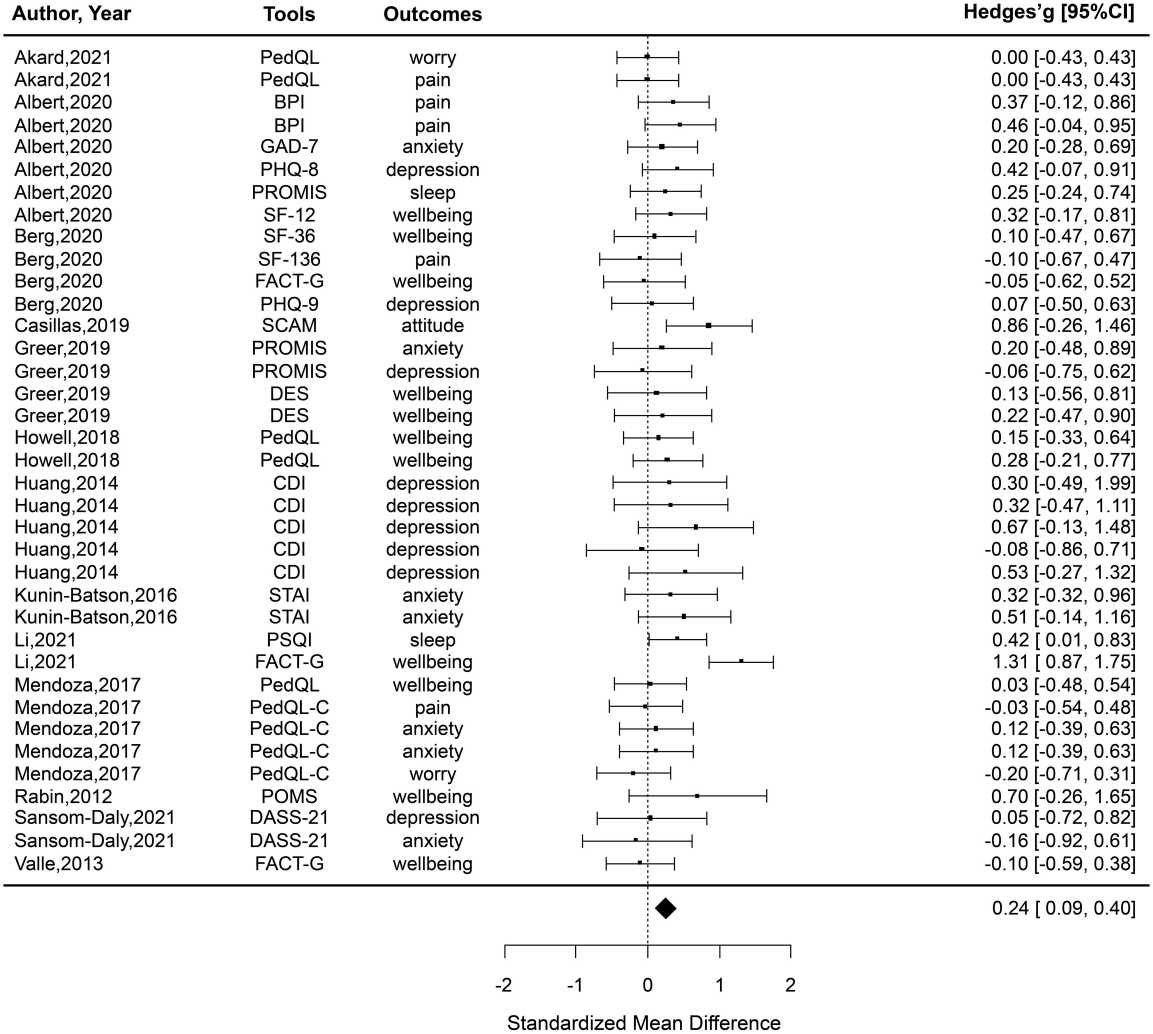
All effect sizes included in the meta-analysis from the studies comparing online interventions to a control group.

A primary analysis compared post-test means of PAYA's mental health outcomes for online intervention and control groups (*n* = 13, *k* = 37; see Figure 3). Only two comparators, *g* = 1.31 and *g* = 0.86, have a statistically significant positive effect size. The 95% CIs for the 35 comparators, including zero, indicated that no statistically significant difference was found between the intervention and control groups. Overall, the pooled effect size was small (*g* = 0.24, 95% CI 0.09–0.40) with low heterogeneity (*I*^2^ = 24%, *Q* = 47.34, *p* = 0.009). A statistically significant difference in favor of the intervention group was found (Z = 3.10, *p* = 0.002) (Figure 3).

#### Analysis of Follow-Up Effectiveness

At the follow-up time points (*n* = 3, *k* = 10; Table 3), we examined the efficacy of the online interventions. The mean scores from the studies' follow-ups were used in this analysis. Two comparators from one study indicated that the intervention groups had statistically significant positive effect sizes ranging from 0.44 to 1.22. However, no statistically significant differences were found between the groups using the eight comparators. The pooled effect size was small (*g* = 0.17, 95% CI −0.40 to 0.74), with moderate heterogeneity (*I*^2^ = 71%) and a non-statistically significant difference (Z = 0.59, *p* = 0.555).

**Table 3:** Sensitivity analysis at post-test.

**Sensitivity analysis**	***g***	**95% CI**	***n***	***k***	***p***	**I^2^**
Follow up	0.17	−0.40 to 0.74	3	10	0.555	71
**1. Age group**						
Children and adolescent	0.10	−0.05 to 0.25	4	14	0.174	0
Young adult	0.31	0.09–0.54	9	23	0.006	39
**2. Outcomes**						
Depression	0.17	−0.13 to 0.47	4	4	0.648	0
Anxiety	0.05	−0.15 to 0.25	5	7	0.922	0
Pain	0.13	−0.13–0.39	4	5	0.416	0
Sleep	0.35	0.04–0.66	2	2	0.595	0
Psychological well-being	0.32	0.09–0.56	10	18	0.032	42
**3. Intervention model**						
CBT	0.04	−0.17 to 0.24	3	10	0.722	0
Psycho-education	0.58	0.19–0.98	2	3	0.004	0
Physical activity enhancement	0.30	−0.01 to 0.61	6	16	0.035	55
Legacy intervention	0.00	−0.30 to 0.30	1	2	0.999	0
**4. Intervention format**						
Individual	0.24	0.11–0.36	9	27	<0.001	0
Group	0.20	−0.27 to 0.67	4	10	0.409	72
**5. Control condition**						
Alternative treatment	0.19	0.02–0.38	7	16	0.048	0
Waiting list	0.19	0.01–0.38	3	12	0.047	0
TAU	0.41	−0.11 to 0.92	3	9	0.122	71
**6. Therapist support**						
Self-directed	0.29	0.10–0.48	10	26	0.003	38
Therapist support	0.11	−0.11 to 0.33	3	11	0.326	0
**7. Platform**						
Website alone	0.19	−0.02 to 0.40	4	7	0.078	0
Website and text message	0.52	0.10–0.94	2	6	0.016	0
Wearable device and social media	0.27	−0.38 to 0.86	3	8	0.379	77
Respiratory monitoring	0.34	0.14–0.54	1	6	0.001	0
Mobile application	0.00	−0.28 to 0.29	1	4	0.976	0
Chatbot	0.12	−0.22 to 0.46	1	4	0.491	0
VDO conference	−0.05	−0.60 to 0.49	1	2	0.846	0

#### Sensitivity Analysis

##### Age Group

Young adult studies (mean age > 20 years) revealed a larger effect size (*g* = 0.31; 95% CI 0.09–0.54) compared to childhood and adolescence studies (*g* = 0.10; 95% CI −0.05 to 0.25).

##### Outcome

A sensitivity analysis was performed to test for variations in effect size between the intervention and control groups' post-treatment scores for depression (*n* = 4), anxiety (*n* = 5), pain (*n* = 4), sleep (*n* = 2), and psychological well-being (*n* = 10). When compared to studies that measured psychological well-being (*g* = 0.32; 95% CI 0.09–0.56), those that used sleep measures had a larger positive effect size (*g* = 0.35; 95% CI 0.04–0.66). Conversely, depression (*g* = 0.17; 95% CI −0.13 to 0.47), anxiety (*g* = 0.05; 95% CI −0.15 to 0.25), and pain (*g* = 0.13; 95 % CI −0.13 to 0.39) had no effect.

##### Intervention Model

A sensitivity analysis was carried out to test for differences in effect size among the intervention and control groups' post-treatment scores, according to the intervention model. Studies using psychoeducational interventions (*n* = 2, *k* = 3) reported a medium effect size (*g* = 0.58; 95% CI 0.19–0.98) with no heterogeneity (*I*^2^ = 0). However, CBT (*n* = 3), physical activity enhancement (*n* = 6), and legacy intervention (*n* = 1) demonstrated a non-significant overall effect with low to medium heterogeneity (*I*^2^ = 0–55).

##### Intervention Format

A sensitivity analysis was conducted to determine whether the effect size of post-treatment effects varied between studies with individual (*n* = 9) and group interventions (*n* = 4). Individual intervention studies had a small effect size (*g* = 0.24, 95% CI 0.11–0.36). There was no statistically significant difference in the overall effect size of group intervention between the intervention and control groups (*g* = 0.20; 95% CI: −0.27 to 0.67) with medium heterogeneity (*I*^2^ = 72).

##### Control Condition

A sensitivity analysis was conducted to determine whether the effect size of post-treatment effects varied between studies with alternative treatment (*n* = 7), waiting list (*n* = 3), and treatment as usual (*n* = 3). Although the biggest effect size is reported for studies comparing treatment-as-usual (*g* = 0.41; 95% CI −0.11–0.92) to alternative treatment (*g* = 0.19; 95% CI 0.02–0.38) and waiting list (*g* = 0.19; 95% CI 0.01–0.38), this subgroup demonstrates no effect.

##### Therapist Support

A sensitivity analysis was conducted to test whether the effect sizes between the intervention and control group's post-treatment scores varied when the intervention group received therapist support. The positive effect size in the self-directed intervention studies was small (*g* = 0.29; 95% CI 0.10–0.48). Nonetheless, no effect was shown when therapist support was used (*g* = 0.11; 95% CI −0.11 to 0.33).

##### Platform

A sensitivity analysis was performed to determine the effect size differences between the intervention and control group post-treatment ratings when using an online platform. A medium effect size (*g* = 0.52; 95% CI 0.10–0.54) with no heterogeneity (*I*^2^ = 0) was found in the studies using websites with text messages (*n* = 2), while no effects were found for studies that only used websites (*g* = 0.19; 95% CI −0.02 to 0.40). A small effect size (*g* = 0.34; 95% CI 0.14–0.54) was found in the studies using respiratory monitoring (*n* = 1). No effects were found for studies that used wearable devices and social media (*g* = 0.27; 95% CI −0.38 to 0.86), mobile application (*g* = 0.00; 95% CI −0.28 to 0.29), chatbots (*g* = 0.12; 95% CI −0.22 to 0.46), and VDO conferences (*g* = −0.05; 95% CI −0.60 to 0.49).

#### Continuous Moderators (Meta-Regression)

The participants' mean age (slope = 0.007, *p* = 0.417), duration of intervention (slope = 0.01, *p* = 0.483), dropout rate (slope = −0.01, *p* = 0.18), and ROB (slope = −0.03, *p* = 0.20) had no effect on the mental health outcomes.

## Discussion

While psychosocial interventions have been recommended to improve the mental health of PAYA cancer survivors (18), few patients obtain them (31). Online interventions are a viable way to broaden access to psychosocial interventions, and trials that support their effectiveness have been published (47–49). The present study conducts a meta-analysis to investigate how online interventions improve the mental health of PAYA cancer survivors. Thirteen studies with a total sample size of 934 participants were included in the total search results. The findings indicate small effect sizes for online intervention groups, with statistically significant differences from control groups, implying that online interventions can improve the mental health of PAYA cancer survivors.

The current findings match and extend the findings of an earlier meta-analysis on a technology-assisted intervention by Zhang et al. (48). However, the effect sizes of this previous meta-analysis were pooled across heterogeneous studies, with varied types of intervention (both online and offline technologies) and objectives (e.g., mental health, cancer knowledge, and distraction of intrusive treatment). In addition to Zhang et al.'s (48) inquiries, we examined the effectiveness of interventions for certain outcomes and intervention delivery methods. These findings have practical implications. Practitioners interacting with families of PAYA cancer survivors may be particularly interested in the efficacy of online therapies tailored specifically to minimize PAYAs' mental health problems.

We evaluated online interventions for PAYA cancer survivors across multiple outcome domains. Online interventions show a significantly small effect size for sleep and psychological well-being. However, the overall intervention effects for depression, anxiety, and pain were non-significant. This is concerning because these outcomes are critical for mental health care services for PAYA cancer survivors, which highlights the importance of developing different interventions to target these mental health symptoms for PAYA. Our findings on psychological well-being outcomes are consistent with previous meta-analyses, which found that a technology-assisted intervention improves psychological well-being (48) but not physical health (47, 48).

Nearly half of the online interventions provided physical activity enhancement as a core intervention model. However, physical activity enhancement demonstrated a non-significant effect on the improvement of PAYA's mental health. A previous meta-analysis showed that a distance-delivered physical activity intervention can improve only physical health-related quality of life but did not increase the physical activity (e.g., Moderate to vigorous physical activity; MVPA) of childhood cancer survivors (47). It can be assumed that enhancing physical and functional health using online interventions did not have a transfer effect on improving mental health.

Psychoeducation showed the greatest treatment effect sizes, while CBT-based interventions showed statistically non-significant effects. Assuming that an online CBT-based intervention cannot be didactic, PAYA cancer survivors may show poor engagement with these formats of interventions, resulting in suboptimal treatment outcomes (62). This demonstrates that it is critical for future research to focus on designing psychological interventions to improve engagement of PAYA cancer survivors.

This study found that the effects of treatment on mental health outcomes vary by age, within the PAYA age range. The effect sizes on mental health were lower in children and adolescent cancer survivors than in young adult cancer survivors. Despite the innovative approach, online intervention contents for adolescents remain insufficient, necessitating the ongoing clinical and research efforts to improve care and mental health outcomes for PAYA cancer survivors specifically. One possible explanation is that most interventions did not provide adolescent-specific materials (61, 63, 64, 68). As a result, online interventions do not adequately meet age-specific needs.

Self-directed or individual online interventions were no less effective than those with therapist support or group-delivered were; however, the small number of interventions may have hampered the detection of existing differences (53). These findings are consistent with those of another meta-analysis (30), which found that in-person and telehealth-involved therapies are equally beneficial for various mental health issues. However, in our findings, when the effect sizes for therapist-led online interventions were pooled across the three studies, and those for group-based online interventions were pooled across the four studies, these findings were inconclusive.

Combining websites with text messages in online interventions can lead to the highest effect on improving mental health outcomes (63, 69), while websites alone showed non-significant effects (61, 65, 68, 72). Despite the effect sizes for websites with text messages pooled across the two studies, a multiple platform (one study using websites with text messages and one using websites with text messages and phone calls) may increase the effect of online interventions; therefore, future studies are needed to confirm the benefit of multimodality online interventions. A novel technology such as wearable respiratory monitoring also showed a significant effect. However, further studies are needed to identify an effective delivery platform. As many interventions in this review used multiple delivery platforms, there is considerable scope to examine the impact of a different platform.

The effects of online interventions on mental health outcomes marginally diminished from posttest to follow-up, according to the follow-up assessments. However, because the effect sizes were aggregated over four investigations, these findings should be interpreted with caution. Although our findings show that the effects wane over time, more research is needed to provide a more detailed explanation.

### Clinical Implications

Although the overall effect of online interventions for improving the mental health of PAYA cancer survivors was promising, health care providers should consider that they are not effective for all outcomes, especially critical outcomes for the improvement of depression, anxiety, and pain. Recommendation for online interventions need to consider for the age group of patients as significant effect was found among interventions for young adults.

### Study Limitations

The first limitation is the study's small sample size. Although Fu et al. (53) recommended a minimum number of studies (at least six for a meta-analysis and four for subgroup analyses of each group), more studies would provide the opportunity for subgroup analyses. As a result, sensitivity analyses were preferred for groups with fewer than four studies. However, if possible, subgroup analyses would have allowed for more specific identification of the intervention characteristics associated with intervention effects. Second, as only three RCTs had a pre-registered protocol, determining whether the data were reviewed according to a pre-defined method was difficult. Thus, practically every study was found to exhibit some amount of bias in the fifth area of RoB 2.0 selection of reported outcomes. Third, all but one study (parent reports) used self-reports to assess mental health outcomes. Therefore, the PAYA cancer survivors were aware of the examination, which may have influenced their mental health scores. Fourth, treatment effects for PAYA survivorship outcomes may differ by age/developmental stages within the PAYA age range. There was a lack of online interventions for children under the age of ten. Online intervention delivered by some delivery methods may be effective for young adults, but not feasible for younger children. The effectiveness of online interventions for children will be determined in future meta-analyses once sufficient papers have been published. Fifth, few studies compare online interventions to traditional interventions (e.g., group-based psychoeducation and in-person CBT). We were unable to determine the efficacy of online vs. traditional therapies on mental health outcomes.

### Future Directions

Self-reports may overstate the effectiveness of interventions (74, 75); thus, future research should utilize more objective tools or parental reports. As mobile phones become the most frequent devices for connecting to the Internet (76), specially designed RCTs should investigate the efficacy of m-health interventions that include the functions of sending reminders, providing feedback, and monitoring (77). The most frequently cited advantage of online interventions is their accessibility (78). Although we discovered that online interventions improve PAYAs' mental health, the pooled effect size was small. Therefore, future studies should find innovative strategies to improve their effectiveness. More studies should compare self-directed online interventions with those provided face-to-face or by videoconference to gain a better understanding of which online interventions are more beneficial. Finally, while this study's objective was not to investigate the mechanisms of change, our findings are consistent with the fundamental assumptions of cancer psychological interventions, which state that increasing cancer knowledge, physical health, and self-efficacy can improve mental health outcomes (79). Future meta-analytic approaches may use a novel methodology, such as meta-analytic structural equation modeling, to provide definitive answers on this subject (80, 81). Thus, it is feasible to evaluate whether gains in physical health, cancer knowledge, psychological skills (CBT or relaxation), or self-efficacy influence the effect of therapies on mental health outcomes.

## Conclusions

Online interventions are effective in improving the mental health outcomes of PAYA cancer survivors. The present study contributes to the body of knowledge offered by prior meta-analyses. Online interventions were found to be effective for sleep and psychological well-being but not for depression, anxiety, or pain. The small number of studies found to be eligible for this meta-analysis, the paucity of RCTs using pre-registered protocols, and outcome measurement dependent on self-reports are all key limitations. Future studies should design more effective interventions targeted at areas related to depression, anxiety, or pain.

## Data Availability Statement

The raw data supporting the conclusions of this article will be made available by the authors, without undue reservation.

## Author Contributions

NC and TT contributed to the design and implementation of the research, to the analysis of the results, and to the writing of the manuscript. All authors contributed to the article and approved the submitted version.

## Conflict of Interest

The authors declare that the research was conducted in the absence of any commercial or financial relationships that could be construed as a potential conflict of interest.

## Publisher's Note

All claims expressed in this article are solely those of the authors and do not necessarily represent those of their affiliated organizations, or those of the publisher, the editors and the reviewers. Any product that may be evaluated in this article, or claim that may be made by its manufacturer, is not guaranteed or endorsed by the publisher.
