# Supplementary material for: Online Interventions to Improve Mental Health of Pediatric, Adolescent, and Young Adult Cancer Survivors: A Systematic Review and Meta-Analysis
Source: Front Psychiatry. 2021 Dec 23;12:784615. doi: 10.3389/fpsyt.2021.784615 (PMC8733740; doi:10.3389/fpsyt.2021.784615)
Supplement: Supplementary file 1 [file Data_Sheet_1.docx]

**Appendix 1**

**Search Online interventions to improve mental health of pediatric, adolescent, and young adult cancer survivors**

**PsycINFO**

*Ovid*

**#1 Cancer**

childhood cancer/ or childhood oncology/ or pediatric cancer/ OR (((child* OR pediatric* OR youth* OR adolescen* OR young OR AYA) ADJ2 (cancer* OR oncology*)) OR childhood cancer survivor* OR AYA cancer survivor* OR young adult cancer survivor*).ti,ab,id.

**#2 Online**

blogging/ OR internet/ OR social media/ OR social networking/ OR Therapy, Computer-Assisted/ OR telemedicine/ OR (Internet* OR computer* OR online OR on-line OR email* OR mail* OR chat* OR web* OR social media OR twitter OR facebook OR youtube OR snapchat OR instagram OR blog* OR digital OR social network* OR whatsapp).ti,ab,id.

**#3 intervention**

(training* OR intervention* OR program*).ti,ab,id.

**1 AND 2 AND 3**

**Medline**

*Ovid MEDLINE(R) Epub Ahead of Print, In-Process & Other Non-Indexed Citations, Ovid MEDLINE(R) Daily and Ovid MEDLINE(R) 1946 to Present*

**#1 Cancer**

childhood cancer/ or childhood oncology/ or pediatric cancer/ OR (((child* OR pediatric* OR youth* OR adolescen* OR young OR AYA) ADJ2 (cancer* OR oncology*)) OR childhood cancer survivor* OR AYA cancer survivor* OR young adult cancer survivor*).ti,ab,kf.

**#2 Online**

blogging/ OR internet/ OR social media/ OR social networking/ OR Therapy, Computer-Assisted/ OR telemedicine/ OR (Internet* OR computer* OR online OR on-line OR email* OR mail* OR chat* OR web* OR social media OR twitter OR facebook OR youtube OR snapchat OR instagram OR blog* OR digital OR social network* OR whatsapp).ti,ab,kf.

**#3 Intervention**

(training* OR intervention* OR program*).ti,ab,kf.

**1 AND 2 AND 3**

**Web of Science**

**#1 Cancer**

TS=((("child*" OR "pediatr*" OR "youth*" OR "adolescen" OR "AYA") NEAR/1 ("cancer*" OR "oncology*" OR "cancer survivor*")) OR "AYA cancer*" OR "young adult cancer*")

**#2 Online**

TS=("internet*" OR "computer*" OR "online" OR "on-line" OR "email*" OR "mail*" OR "chat*" OR "web*" OR "social media" OR "twitter" OR "facebook" OR "youtube" OR "snapchat" OR "instagram" OR "blog*" OR "digital" OR "social network*" OR "whatsapp")

**#3 intervention**

TS=("training*" OR "intervention*" OR "program*")

**1 AND 2 AND 3**

**Cochrane Library**

**#1 Cancer**

(("child*":ti,ab,kw OR "pediatr*":ti,ab,kw OR "youth*":ti,ab,kw OR "adolescen":ti,ab,kw) NEAR/1 ("cancer*":ti,ab,kw OR "oncology*":ti,ab,kw OR "cancer survivor*":ti,ab,kw)) OR " AYA cancer *":ti,ab,kw OR "young adult cancer*":ti,ab,kw

**#2 Online**

"internet*":ti,ab,kw OR "computer*":ti,ab,kw OR "online":ti,ab,kw OR "on-line":ti,ab,kw OR "email*":ti,ab,kw OR "mail*":ti,ab,kw OR "chat*":ti,ab,kw OR "web*":ti,ab,kw OR "social media":ti,ab,kw OR "twitter":ti,ab,kw OR "facebook":ti,ab,kw OR "youtube":ti,ab,kw OR "snapchat":ti,ab,kw OR "instagram":ti,ab,kw OR "blog*":ti,ab,kw OR "digital":ti,ab,kw OR "social network*":ti,ab,kw OR "whatsapp":ti,ab,kw

**#3 intervention**

"training*":ti,ab,kw OR "intervention*":ti,ab,kw OR "program*":ti,ab,kw

**1 AND 2 AND 3**

**Appendix 2**

**Included studies**

1. Akard TF, Dietrich MS, Friedman DL, Wray S, Gerhardt CA, Hendricks-Ferguson V, et al. Randomized clinical trial of a legacy intervention for quality of life in children with advanced cancer. J Palliat Med 2021;24(5):680-688.
2. Alberts NM, Leisenring WM, Flynn JS, Whitton J, Gibson TM, Jibb L,et al. Wearable respiratory monitoring and feedback for chronic pain in adult survivors of childhood cancer: a feasibility randomized controlled trial from the childhood cancer survivor study. JCO Clin Cancer Inform 2020;4:1014-1026.
3. Berg CJ, Vanderpool RC, Getachew B, Payne JB, Johnson MF, Sandridge Y, et al. A hope-based intervention to address disrupted goal pursuits and quality of life among young adult cancer survivors. J Cancer Educ 2020;35(6):1158-1169.
4. Casillas JN, Schwartz LF, Crespi CM, Ganz PA, Kahn KL, Stuber ML,et al. The use of mobile technology and peer navigation to promote adolescent and young adult (AYA) cancer survivorship care: results of a randomized controlled trial. J Cancer Surviv 2019;13(4):580-592.
5. Greer S, Ramo D, Chang YJ, Fu M, Moskowitz J, Haritatos J. Use of the chatbot "Vivibot" to deliver positive psychology skills and promote well-being among young people after cancer treatment: randomized controlled feasibility trial. JMIR Mhealth Uhealth 2019;7(10):e15018.
6. Howell CR, Krull KR, Partin RE, Kadan-Lottick NS, Robison LL, Hudson MM, et al. Randomized web-based physical activity intervention in adolescent survivors of childhood cancer. Pediatr Blood Cancer 2018;65(8):e27216.
7. Huang JS, Dillon L, Terrones L, Schubert L, Roberts W, Finklestein J, et al. Fit4Life: a weight loss intervention for children who have survived childhood leukemia. Pediatr Blood Cancer 2014;61(5):894-900.
8. Kunin-Batson A, Steele J, Mertens A, Neglia JP. A randomized controlled pilot trial of a Web-based resource to improve cancer knowledge in adolescent and young adult survivors of childhood cancer. Psychooncology 2016;25(11):1308-1316.
9. Li L, Wang L, Sun Q, Xiao P, Duan Y, Liu X, et al. Effect of two interventions on sleep quality for adolescent and young adult cancer survivors: a pilot randomized controlled trial. Cancer Nurs 2021 Apr 21. doi: 10.1097/NCC.0000000000000932. Online ahead of print.
10. Mendoza JA, Baker KS, Moreno MA, Whitlock K, Abbey-Lambertz M, Waite A, et al. A Fitbit and Facebook mHealth intervention for promoting physical activity among adolescent and young adult childhood cancer survivors: A pilot study. Pediatr Blood Cancer 2017;64(12).
11. Rabin C, Dunsiger S, Ness KK, Marcus BH. Internet-based physical activity intervention targeting young adult cancer survivors. J Adolesc Young Adult Oncol 2011;1(4):188-194.
12. Sansom-Daly UM, Wakefield CE, Ellis SJ, McGill BC, Donoghoe MW, Butow P, et al. Online, group-based psychological support for adolescent and young adult cancer survivors: results from the recapture life randomized trial. Cancers (Basel) 2021;13(10):2460.
13. Valle CG, Tate DF, Mayer DK, Allicock M, Cai J. A randomized trial of a Facebook-based physical activity intervention for young adult cancer survivors. J Cancer Surviv 2013;7(3):355-68.

**Appendix 3**

**PRISMA 2020 checklist**
